# Reconciling Oxygen and Aerosol Delivery with a Hood on In Vitro Infant and Paediatric Models

**DOI:** 10.3390/pharmaceutics14010091

**Published:** 2021-12-31

**Authors:** Shu-Hsin Chen, Hsiu-Chu Chang, Ming-Yi Chien, Jinxiang Xi, Hui-Ling Lin

**Affiliations:** 1 Department of Respiratory Therapy, Keelung Chang Gung Memorial Hospital, Keelung 20401, Taiwan; fatblackcherry@gmail.com (S.-H.C.); hsiuchu8900@gmail.com (H.-C.C.); ming1234@cgmh.org.tw (M.-Y.C.); 2 Department of Biomedical Engineering, University of Massachusetts, Lowell, MA 01854, USA; 3 Department of Respiratory Therapy, Chang Gung University, Taoyuan 33302, Taiwan; 4 Department of Respiratory Care, Chang Gung University of Science and Technology, Chiayi 61363, Taiwan

**Keywords:** aerosol therapy, oxygen therapy, facemask, hood, placement, enclosure system

## Abstract

This study aimed to evaluate optimal aerosol and oxygen delivery with a hood on an infant model and a paediatric model. A facemask and a hood with three inlets, with or without a front cover, were used. A small-volume nebuliser with a unit-dose of salbutamol was used for drug delivery and an air entrainment nebuliser was used to deliver oxygen at 35%. Infant and paediatric breathing patterns were mimicked; a bacterial filter was connected to the end of a manikin trachea for aerosol drug collection, and an oxygen analyser was used to measure the oxygen concentration. For the infant model, inhaled drug dose was significantly higher when the nebuliser was placed in the back of the hood and with a front cover. This was verified by complementary computational simulations in a comparable infant-hood model. For the paediatric model, the inhaled dose was greater with a facemask than with a hood. Oxygen delivery with a facemask and a hood with a front cover achieved a set concentration in both models, yet a hood without a front cover delivered oxygen at far lower concentrations than the set concentration.

## 1. Introduction

Drug delivery through the inhalation route is preferred for the treatment of asthma in children. The Global Initiative for Asthma stated that aerosol therapy plays an important role in children with asthma during the diagnostic process, alleviation of acute exacerbation, and maintenance of airway stability [1,2]. The administration of aerosol and oxygen to hospitalised children can often be complicated by general discomfort and irritability caused by the disease and medical procedures. The structure of the upper airway and the breathing pattern is comparatively distinct in children aged <18 months, with a proportionately large tongue, narrower larynx, and shorter turbinate contributing to airway resistance [3,4]. Additionally, children tend to have shorter inspiration times, irregular and fast respiratory rates, and small tidal volumes, resulting in increased aerosol impaction to the upper airways and lower lung deposition [3,5].

In children, the actual dose delivered by aerosolised drugs is a small proportion of the initial dose, approximately 1.6%–4.4% compared to 10−58% in adults [5]. A previous study showed that delivering aerosol medication during crying and distress greatly decreased drug inhalation to the lungs to only 0.35%−0.7% of the initial dose [6]. Blow-by therapy is an alternative to a tight facemask for delivering aerosol medications to an irritated child. However, the ineffectiveness of aerosol therapy with an ill-fitted facemask has been demonstrated; thus, blow-by treatment is not advised for infants [7,8,9].

An enclosure system, such as a hood, is recommended for oxygen therapy in children less than three years of age [9]. Though, some cases require simultaneous oxygen therapy and aerosol delivery. Amirav et al. administered aerosol through a facemask or a hood to wheezing children aged 1 to 19 months and found similar lung doses between the delivery methods [10]. Children also showed greater tolerance and parents showed greater preference for treatment delivery using the hood. Therefore, the use of a hood has been proposed as a viable option for aerosol therapy in children [11].

Hood designs vary in size, placement of the gas source inlet, and degree to which it is sealed [12]. The optimal design of a hood has not been sufficiently evaluated. The aim of this in vitro study was to evaluate the efficiency of a hood with different gas inlet placements and enclosure systems for oxygen/aerosol therapy.

## 2. Materials and Methods

### 2.1. Experimental Apparatus

A breath simulator (ASL 5000, IngMar Medical Inc., Pittsburgh, PA, USA) was set to mimic a spontaneously breathing infant with tidal volume 50 mL, inspiratory time 0.3 s, peak inspiratory flow 13.1 L/min with a sinusoidal waveform breathing pattern, respiratory rate 33 breath/min, airway resistance 5.04 cm H_2_O/L/s, and compliance 120 mL/cm H_2_O. To represent a spontaneously breathing infant/young toddler between 3–4 years old, the simulator was set to tidal volume = 100 mL, inspiratory time = 0.64 s, inspiratory flow = 14.5 L/min, respiratory rate = 24 breath/min, airway resistance = 5.04 cm H_2_O/L/s, and compliance = 120 mL/cm H_2_O [13,14]. The simulator was connected to the trachea of an infant or paediatric intubation manikin via a corrugated tube whereas aerosol and oxygen were inhaled through the mouth and nose.

**Figure 1:**
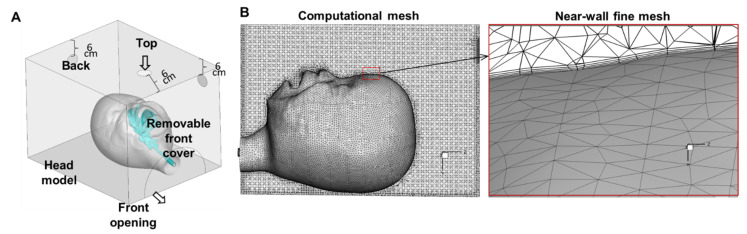
Computational model for drug delivery to a seven-month-old model using a hood: a face-mouth-nose-throat geometry placed in a hood with a removable front cover (**A**); fine computational mesh with five layers of near-wall prismatic mesh (**B**).

To determine optimal oxygen and aerosol delivery, we used a paediatric facemask and two custom-designed oxygen hoods with the following specifications: 22 cm × 20 cm × 25.5 cm for infants and 36 cm × 27 cm × 33 cm for paediatric patients. Holes were made 6 cm for the edge of top, side, and back panels to evaluate the impact of the gas source placement (Figure 1A). A front removable cover was designed to test the influence of appropriate sealing.

### 2.2. Evaluation of Aerosol Delivery

A unit-dose of salbutamol sulfate (5.0 mg/2.5 mL, GlaxoSmithKline, Victoria, Australia) was diluted in 4 mL saline and was placed in a pneumatic-powered nebuliser (50 psi oxygen at the airflow rate of 8 L/min until 2 min after sputter sounded, Neb-Easy, Galemed Corp., Taipei, Taiwan). To reduce the percentage of drug dose remaining the medication cup, salbutamol was diluted before the nebulisation [15]. The nebuliser was connected to the manikin with either a facemask or a hood with three different inlet locations, with or without the front cover. The aerosolised drug was collected using a bacterial filter placed between the end of the trachea of the manikin and the breath simulator. Each nebulisation procedure was repeated five times.

The drug deposits on the collecting filter and drug remaining in the nebuliser (as residual dose) were eluted, and the drug on the face of the manikin was washed with 10 mL of distilled water for 3 min. The absorbance of each drug sample was measured using an ultraviolet spectrophotometer (Thermo Fisher Scientific Inc., Waltham, MA, USA) at a wavelength of 276 nm. There was a linear relationship between the absorption and concentration of salbutamol between 2.0 and 250 µg/mL with a slope of 0.0062 (R^2^ = 0.9999). The salbutamol drug mass was then calculated from the absorption-concentration standard curve.

### 2.3. Evaluation of Oxygen Delivery

**Figure 2:**
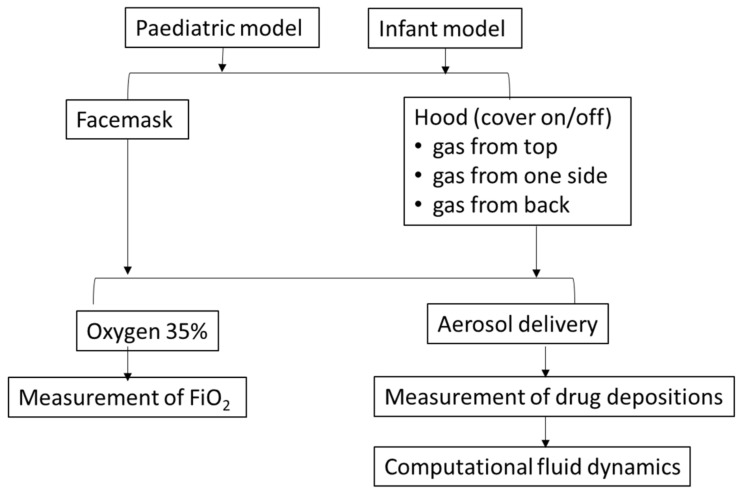
Flow chart of study protocol.

Oxygen was generated by a large-volume nebuliser with an oxygen flow rate set at 10 L/min and a 35% fraction of inspired oxygen (FiO_2_). The total gas flow rate was 40 L/min. Oxygen was delivered through a large-bore corrugated tube to either a facemask or hood, with or without a front cover. An oxygen analyser (Ohio Medical Cooperation, Gurnee, IL, USA) was placed between the end of the bronchi and the breath simulator of the manikin and was calibrated before each test. The oxygen concentration was recorded within variations of ± 0.1% for 30 s, and the time to reach a stable concentration was recorded. Each experiment was repeated five times. Figure 2 presents the flow chart of study protocol for quantification of oxygen concentration and aerosol drug dose.

### 2.4. Numerical Analysis

To assess the flow and particle dynamics in the hood delivery system, numerical simulations were conducted using a seven-month-old infant model within a hood with three respective nebuliser positions (Figure 1). The hood dimension, the relative position of the infant head model, and the three nebuliser positions followed the infant experimental set up.

The computational infant head model consisted of the face, mouth, nose, and pharyngolaryngeal airway (or throat), originally reconstructed from CT scans of a seven-month-old female (height 71 cm and weight 9.3 kg) [16]. The volume geometry of the face-mouth-nose-throat airway was subtracted from the hood volume, yielding the airspace within the hood and the respiratory tract (Figure 1A). The openings for the nebuliser, the removable front cover of the hood, and the front opening of the hood could be configured as inlet/outlet or wall to simulate different nebuliser positions with or without a cover (Figure 1A). ANSYS ICEMCFD (Ansys Inc., Ann Arbor, MI, USA) was used to generate the computational mesh. To accurately resolve the flow transitions near the wall, five layers of body-fitted prismatic mesh were generated in the near-wall region, with the height of the first layer being 0.05 mm (Figure 1B). A grid-independent study was performed by changing the total mesh size to 1.2, 2.4, 4.0, 6.4, and 9.0 million cells. Grid-independent results were achieved at the mesh size of 6.4 million cells, with less than 1% variation in the delivered fraction between 6.4 and 9.0 million cells. The mesh size of 6.4 million cells was consequently selected for all subsequent simulations.

ANSYS Fluent was employed to elucidate airflow and track particle motions. Airflows were assumed to be Newtonian flows, isothermal, and incompressible. Only steady inhalations were simulated to assess particle movement during peak inhalation. Based on its capacity in capturing laminar-turbulent transitions, a low-Reynolds-number (LRN) *k-ω* turbulence model was used to assess the flow field [17]. Particle trajectories were tracked using a discrete-phrase Lagrangian approach that was enhanced with user-defined functions (UDFs) of near-wall treatment for velocity and turbulence anisotropy [18]. The combined LRN *k-ω* turbulence model and UDF-enhanced Lagrangian approach have been sufficiently validated in previous studies with good agreement with complementary experiments for both nano- and micrometre-sized particles [19,20,21]. The opening for the nebulised aerosol was specified as the inlet, with a speed of 0.272 m/s (or 8.0 L/min). The inhalation flow rate was 13.0 L/min for the infant model to simulate the peak inhalation. The average median mass aerodynamic diameter for tested nebulisers was 4.51 µm, as tested using a Next Generation Impactor (Copley Scientific, Nottingham, UK) [22]. Second-order spatial discretisation or higher was used for all transport terms. Convergence of the airflow solution was achieved when the mass residual decreased by five orders of magnitude and the residual variation profiles for both mass and momentum plateaued.

### 2.5. Statistical Analysis

The drug dose was deposited on the filter and face, and the residual dose were expressed as a percentage of the initial loaded dose (5.0 mg salbutamol). One-way analysis of covariance was conducted with Tukey’s post hoc tests. Independent *t*-tests were used to analyse differences between the use of a cover and without a cover. Data were analysed using the SPSS version 26.0 (IBM Inc., New York, NY, USA). Statistical significance was set at *p* < 0.05.

## 3. Results

### 3.1. Aerosolised Drug Deposition

Table 1 presents the percentage drug deposition of the delivered dose, dose deposited on the face, and residual dose. With the infant model, the dose was most effectively delivered from the back of the hood with a front cover attached (*p* < 0.005). In the paediatric model, drug dose delivery was significantly higher through the facemask than the hood, in all experimental conditions (*p* < 0.001). For both paediatric and infant models, drug deposition to the face was similar between the facemask and the top inlet of the nebuliser in the hood and was significantly lower with nebuliser inlets at the side or back of the hood without a front cover (*p* < 0.001). The residual dose in the nebuliser was similar across all the conditions.

### 3.2. Numerical Analysis of Hood Delivery

**Figure 3:**
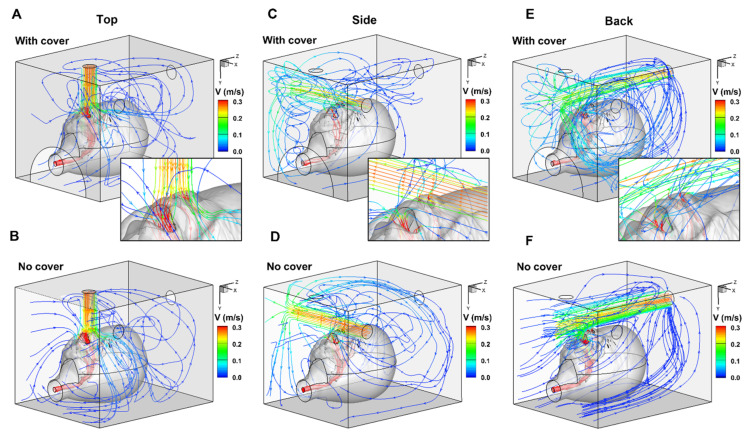
Airflow streamlines of hood delivery to a seven-month-old model with three nebuliser positions: top (**A**,**B**), side (**C**,**D**), and back (**E**,**F**), with (**A**,**C**,**E**) or without (**B**,**D**,**F**) the front cover with visualised using different colours. The inset in (**A**,**C**,**E**) shows the inspiratory flow around the mouth and nose openings.

**Figure 4:**
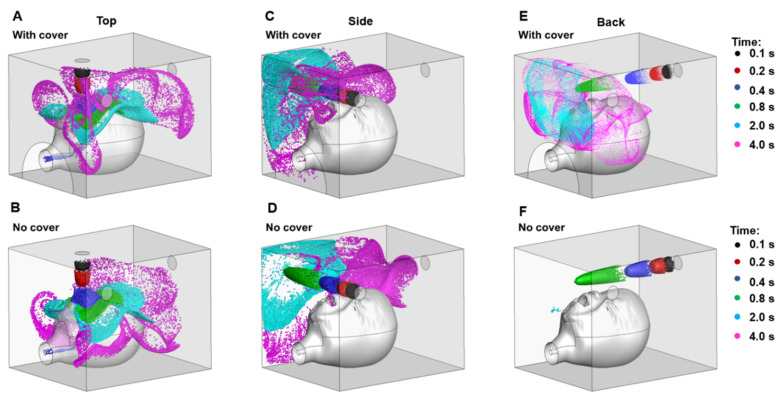
Snapshots of particle positions at varying increments of time after release in the hood delivery system with three nebuliser positions: top (**A**,**B**), side (**C**,**D**), and back (**E**,**F**), with (**A**,**C**,**E**) or without (**B**,**D**,**F**) the front cover.

Figure 3 and Figure 4 illustrate the patterns of hood delivery to the infant model with three nebuliser positions, each with or without the front cover. The flow patterns differed between each of the six scenarios. Attachment of the front cover created recirculation flow in the hood (Figure 3A,C,E), which was significantly reduced in the absence of the front cover (Figure 3B,D) and was not observed with the back nebuliser position (Figure 3F). Recirculation flow can increase the residence time of particles within the hood, increasing the likelihood of a dose particle being inhaled and successfully delivered.

The effect of the nebuliser position can be seen in the extent of recirculation flow. The top inlet creates a recirculation zone mainly above the head (Figure 3A), while the side inlet creates a recirculation zone near the opposite wall (Figure 3C). This is expected as the head and the opposite wall are the blockages where the nebuliser flows opposite to their direction. With the back inlet, two recirculation zones were observed near the two side walls (Figure 3E). The extent of these recirculation zones was relatively large, as the stream traces travel back and forth along the long side of the hood. The programmed inhalation rate (13.0 L/min) was higher than the nebuliser flow rate (8.0 L/min), leading to a compensation flow of 5.0 L/min into the hood from the front opening. This compensation flow should further promote recirculation flow along the length of the hood (i.e., z direction, Figure 3E). The local flow patterns around the mouth and nose openings are shown in the insets of Figure 3A,C,E.

Figure 4 shows the snapshots of aerosol clouds at varying increments of time after release, i.e., 0.1, 0.2, 0.4, 0.8, 2.0, and 4.0 s, which are visualised using different colours. Cloud shape varied depending on the inlet position and the presence of a front cover (Figure 4). Aerosols released from the top were distributed above and around the head (Figure 4A,B). The cloud gradually settled toward the bottom in the absence of a front cover (Figure 4B). With a front cover, the clouds became more ambient due to a strong compensation flow (Figure 4A). Aerosols released from the side wall were concentrated near the opposite side wall, and the presence of a front cover reduced the escape of particles from the upper-front-left corner (Figure 4C,D). The most pronounced difference was observed when aerosols were released through the back wall. With the blockage of the front cover, particles concentrated to the front-upper-middle area of the hood, where particles have a greater chance of being inhaled (Figure 4E). The cloud was also more dispersed than that released from the top and side, indicating an elevated recirculation in this region. As expected, aerosols were more likely to escape when the front cover was absent (Figure 4F).

**Figure 5:**
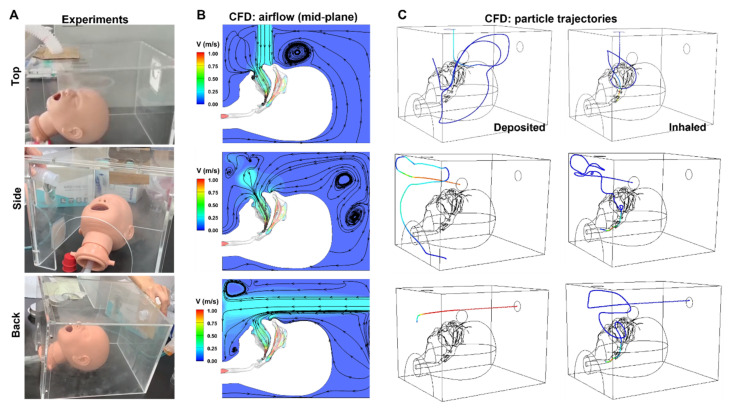
Experiments (**A**) vs. computational fluid dynamic (CFD) predictions: mid-plane contour of airflow speed (**B**) and trajectories of sample particles that are either deposited on the wall or being inhaled into the mouth (**C**).

Figure 5 shows the experimental photos (Figure 5A) vs. CFD predictions of airflow (Figure 5B) and particle trajectories (Figure 5C). Overall, the CFD-predicted airflows confirmed our observations of the nebulised aerosol plumes. Particularly, the flow recirculation above the infant’s forehead that was observed with the top nebuliser position was also captured in CFD predictions (upper row, Figure 5A,B). Figure 5C shows the motions of sample particles that are either deposited on the wall of the hood or being inhaled into the mouth. For most particles, tortuous trajectories were predicted in the hood, reflecting the complex aerosol dynamics during hood nebulisation.

### 3.3. Oxygen Therapy

The measured FiO_2_ for both models reached the set FiO_2_ within 5 min (*p* > 0.05). Table 2 shows oxygen concentration stabilisation time between mask and hood delivery in infant and paediatric models. In the infant model, the FiO_2_ was significantly lower than the set FiO_2_ when oxygen was provided through the side and back of the hood without a front cover (*p* < 0.0001). In the paediatric model, the set FiO_2_ was only reached using a hood with a front cover; the FiO_2_ was slightly lower with a facemask and significantly lower with a hood without a front cover.

## 4. Discussion

The present study demonstrated that an optimal aerosol delivery system for the infant model consisted of a well-sealed hood with a nebuliser inlet at the back panel, though a facemask proved optimal for dose delivery to a paediatric model. In both models, oxygen delivery was equally effective between a hood with a front cover and a facemask. Irrespective of the physical specifications, a hood without a front cover could not provide a sufficient oxygen concentration to the paediatric model.

A facemask or hood is the recommended interface for aerosol therapies in children aged 1−3 years [11]. A facemask is commonly used as an interface for a nebuliser during aerosol therapy. However, the commercially available facemasks are limited in design and do not suitably fit children of different ages. Aerosol loss in standard paediatric facemasks can be attributed to the ventilation holes and inadequate seal around the mouth and nose, leading to a decrease in the medication available to the patient [14]. Studies have shown similar aerosol-delivered drug doses between hoods and facemasks, though children are more tolerant of treatments with the hood [10,23,24]. Our results showed that aerosol delivery with a facemask delivered a greater dose in the paediatric model, but was less effective in the infant model.

### 4.1. Aerosol Therapy with a Facemask vs. a Hood in an Infant Model

A disproportionately large facemask can be an irritation to the infant, while only a few facemask sizes are available for various face sizes in infants. In addition, the leaked flow from the gaps between the mask and face can carry the aerosol toward the eyes of the infant through the noise, thus easily irritating the eyes; the external flow at 8 L/min carries the aerosol toward the eyes of an infant patient [25,26].

Our results showed that optimal drug delivery in a paediatric model was achieved with the facemask. The delivered doses using the hood were similar when the nebuliser being placed at the back or top with a front cover. The delivered drug dose through the hood was lower without a front cover for all three nebuliser positions (Table 1). The aerodynamics inside the hood greatly impacted the availability of drugs to the mouth and nose during respiration. When the nebuliser was placed at the back of the hood with a front cover, the cloud covers the infant’s forehead up to the chin. The cloud was then reflected by the front cover of the hood, creating a continuous circulation flow surrounding the head (lower panels, Figure 5A–C). According to Bernoulli’s principle, the aerosol flow on the forehead pushes the circulating flow to the infant’s face, increasing the chance of the aerosol being inhaled. Kim et al. studied head positions and two breathing models using a computational fluid dynamics approach and found that the inhalation of aerosol particles was dependent on the dynamic rivalry between gravity-related sedimentation and the drag force created by the inhalation flow toward the mouth and nose [16].

Without the front cover and a properly sealed hood, a portion of the aerosol cloud was lost and the delivered drug dose was reduced (left lower panel, Figure 5C). This study also showed that the inlet in the back panel was better positioned at a height greater than the infant’s head. This avoids the immediate blockage to flow that the upper head would create and allows for a gradual downward trajectory of aerosol flow towards the mouth/nose openings.

**Figure 6:**
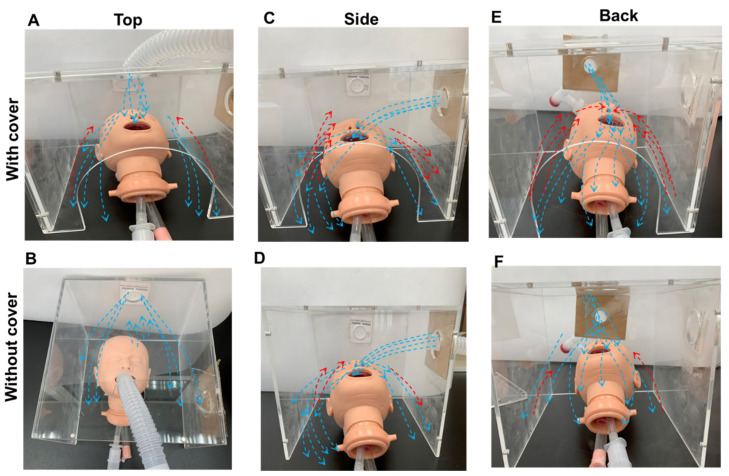
Aerosol delivery using a hood with a nebuliser placed at three positions: the side (**A**,**B**), back (**C**,**D**), and top (**E**,**F**), with (**A**,**C**,**E**) or without (**B**,**D**,**F**) a cover. The observed aerosol clouds are marked as blue lines for incoming aerosol clouds and red lines for bounced aerosol clouds.

Kim et al. found a greater proportion of dose inhaled and lower levels of facial deposition when positioning an infant closer to the side of the hood [16]. When the nebuliser was placed at the side, aerosol clouds flowed in two directions after encountering the internal wall of the hood: one direction was toward the back of the hood, circulating around the head, and the other toward the front and outside of the hood (Figure 3B and Figure 6A). Additionally, a small proportion of the aerosol was deposited on the face. With a cover, the aerosol cloud bounced back toward the face of the infant, so that the inhaled dose was slightly lower compared with the dose flowing through the back inlet (Figure 5C and Figure 6A). Amirav et al. found that the configuration of the hood design could reduce aerosol delivery efficiency, particularly when the funnel, head, or both were tilted [27].

When placing the nebuliser on the top (Figure 6E), the corrugated tube was positioned at an inclined angle to keep the nebuliser at a vertical position; thus, aerosols directly flowed to the nose and forehead, resulting in a greater drug deposition on the face similar to that of a facemask (upper panels, Figure 5A,B). For the treatment of an infant, a nebuliser placed at the back of the hood was more effective than a facemask (Figure 6C). For all three nebuliser positions, a higher delivered dose was achieved with a front cover compared to without. Therefore, when using a hood to administer an aerosol, it is recommended that the hood be sealed for optimal dose delivery.

The amount of drug deposition on the face was similar and considerably high (3.18%–4.55%) between the facemask and the hood with the nebuliser placed on the top or at the back. The amount of aerosol deposited on the face was lower when the nebuliser was placed on the side than at the other positions. The high dose deposited on the face may have potential risks to the local bacterial balance when using nebulised corticosteroids or antibiotics. Facial cleaning is encouraged after nebulisation treatment regardless of the interface used.

### 4.2. Aerosol Therapy with a Facemask vs. a Hood in a Paediatric Model

In the paediatric model, a higher drug dose was delivered with a facemask than through a hood, regardless of the nebuliser position. It is presumed that a better fitting facemask would reduce the escape of aerosols in toddlers and would allow for more effective delivery of the dose. The aerosol delivery system is designed to fill the dead space of the facemask, thus maximising the dose delivered in each breath. Additionally, a larger tidal volume with a sufficient reservoir system, such a facemask or hood, can contain a greater dose than the infant model could inhale. Shakked et al. used a three-dimensional numerical simulation to test aerosol drug delivery in infants with an innovative hood design and found that increasing tidal volume increased the amount of inhaled particles [26,28]. Moreover, they found that flow circulations inside the hood significantly influenced the motion of particles, resulting in greater deposition on the surface rather than being inhaled, a phenomenon that we also observed (Figure 3, Figure 4 and Figure 5). It is therefore imperative that clinical efficacy be closely monitored in aerosol administration to toddlers using a hood.

### 4.3. Oxygen Concentration

Oxygen therapy is often administered to hospitalised paediatric patients via a hood. A facemask is an alternative interface for children receiving oxygen therapy, though they can be hard to secure to the patient’s face and can cause irritation [29,30]. The efficiency of oxygen delivery greatly depends on the degree to which the hood is sealed. In this study, oxygen delivery was effectively achieved within 5 min through a facemask or a hood with a front cover. Without a cover, the desired oxygen concentration could not be delivered with a hood. 

**Figure 7:**
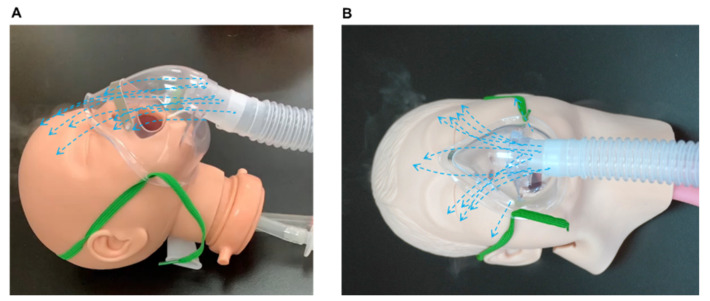
Oxygen delivery with a loosely fit facemask in an infant model (**A**) and tightly fit facemask in a paediatric model (**B**).

We loosely fitted a facemask to an infant model during pilot experiments and found that the FiO_2_ was only 30.6% (Figure 7A). When the face of the infant model was smaller than the facemask, the facemask naturally tilted to a 45° angle, resulting in a greatly reduced reservoir effect and consequently reduced FiO_2_. Our formal experiments utilised the facemask with a tight fit only (Figure 7B). However, this would provide a patient with great discomfort and would therefore be clinically impractical; thus, a well-sealed hood is recommended.

In both models, our results showed that the desired FiO_2_ was achieved by placing the gas source on top of the hood with a front cover. Gas momentum affects the delivered FiO_2_ by disturbing the available gas around the child’s face, and the forward movement of gas flow generates turbulence after encountering an object. When oxygen is administered through the hood with a front cover, the gas moves forward and the turbulent flow concentrates oxygen around the head, resulting in a stable availability of oxygen regardless of nebuliser placement. However, without a front cover, oxygen escapes from the hood, especially when it is introduced through the back panel (Figure 3F).

### 4.4. Study Limitations

We have recognised a few unavoidable limitations to this study. Previous studies have shown that aerosol drug doses significantly decreased when children moved or were upset during the aerosol treatment [6]. Our study, using still models, might therefore overestimate the delivered drug dose compared to a clinical situation. The recovered dose from face, inhaled dose, and residual dose accounted for approximately 60%. It is speculated that the remaining 40% of the drug was deposited on the hood surface, table under the manikin head, and airways of the manikin, or escaped to the air. Due to the open system of the set-up and large contact surface area, we were unable to fund a 100% recovery. Notably, previous studies have also reported 50%–60% of residual dose, which is similar to our results [31,32]. Infants are obligate nose breathers because of a large tongue creating high resistance. However, our infant manikin and the CFD simulation model were designed with a small tongue; thus, air was inhaled largely through the mouth.

The limitations of numerical modelling include using a single-head model, steady inhalation only, and a uniform aerosol size distribution. Considering the effect of cyclic breathing on particle behaviour, the region affected by inhalation was relatively small in the hood, which was located immediately above the mouth/nose openings (Figure 5B). Thus, we have concluded that cyclic breathing would only affect particle dynamics close to the mouth/nose openings, and that using steady inhalation can capture the dominant behaviors of the nebulised aerosols in the hood. Moreover, the use of steady flows greatly simplified numerical simulations. However, to more accurately characterise the aerosol dynamics in the vicinity of the face, cyclic breathing should be considered in future studies. The aerosol droplets have a polydisperse size distribution as opposed to a uniform diameter [33]. Other droplet properties that were possibly present in the experiments, but excluded from the numerical analyses, include sedimentation, electrostatic charge, collision, evaporation, and hygroscopic growth [34,35,36,37,38]. Notably, both experiments and numerical analyses used a stationary head model, which is not representative of the compliant and dynamic airways in real-life situations [39].

## 5. Conclusions

Optimal aerosol delivery was achieved by positioning a nebuliser inlet through the back of a hood with a sealed front cover in an infant model, and with a facemask in a paediatric model. The desired oxygen concentration can be delivered with a facemask or an appropriately sealed hood. Our study demonstrated that a hood without a proper seal and cover failed to deliver optimal oxygen and aerosol medication and is not recommended in practice.

## Figures and Tables

**Table 1:** Drug depositions between the mask and hood delivery systems in infant and paediatric models.

Model	Placement	Deposition (%)		
		Facemask	With Cover	No Cover
Infant				
Inhaled		1.11 ± 0.22		
	Top		1.03 ± 0.09	0.84 ± 0.1
	Side		1.13 ± 0.17	0.89 ± 0.14
	Back		1.68 ± 0.27 *^,†^	1.2 ± 0.13
Face		4.15 ± 0.26		
	Top		4.31 ± 0.29	4.55 ± 0.51
	Side		2.34 ± 0.39 ^†^	1.67 ± 0.2
	Back		4.39 ± 0.61 ^†^	3.18 ± 0.23
Residual dose		50.1 ± 3.22		
	Top		51.98 ± 4.55	52.42 ± 4.53
	Side		51.36 ± 5.09	50.45 ± 4.67
	Back		50.37 ± 3.84	50.85 ± 3.74
Paediatric				
Inhaled		1.89 ± 0.17 *		
	Top		1.37 ± 0.19	1.13 ± 0.17
	Side		1.22 ± 0.24	1.07 ± 0.17
	Back		1.37 ± 0.27	1.13 ± 0.18
Face		4.71 ± 0.43		
	Top		4.42 ± 0.64 **	4.29 ± 0.55
	Side		1.67 ± 0.3 ^†^	1.45 ± 0.17
	Back		2.47 ± 0.29 ^†^	2.26 ± 0.34
Residual dose		48.47 ± 2		
	Top		46.41 ± 3.79	47.82 ± 4.6
	Side		48.94 ± 4.92	48.78 ± 2.8
	Back		50.51 ± 6.65	49.82 ± 5.29

Values expressed as mean ± SD. * *p* < 0.001 greater inhaled dose compared to facemask versus hood. ** *p* < 0.001 greater face deposition compared with side and back. ^†^ *p* < 0.001 comparing covered versus non-covered hoods.

**Table 2:** FiO_2_ and stabilisation time between mask and hood delivery systems in infant and pediatric models.

Model			With Cover	No Cover
Infant				
FiO_2_ (%)	Facemask	35.89 ± 0.12 *		
	Top		35.7 ± 0.14	35.48 ± 0.16
	Side		35.17 ± 0.23 ^†^	26.8 ± 0.26
	Back		35.27 ± 0.25 ^†^	23.72 ± 0.26
Stabilisation time, min				
	Facemask	4.82 ± 0.14		
	Top		3.77 ± 0.6	4.11 ± 1.01
	Side		4.91 ± 0.65	5.03 ± 0.87
	Back		5.03 ± 0.82	4.47 ± 0.6
Paediatric				
	Facemask	33.97 ± 0.15 *		
	Top		35.22 ± 0.17 ^†^	29.89 ± 0.2
	Side		34.83 ± 0.12 ^†^	25.82 ± 0.17
	Back		35.4 ± 0.14 ^†^	23 ± 0.14
Stabilisation time, min				
	Facemask	3.8 ± 0.57		
	Top		4.41 ± 0.34	4.09 ± 0.47
	Side		4.72 ± 0.27	4.57 ± 0.72
	Back		4.36 ± 0.37	4.71 ± 0.79

Values are mean ± SD. * *p* < 0.001 compared with mask versus hood without a cover. ^†^ *p* < 0.001 compared with mask versus hood without a cover.

## Data Availability

The datasets used and/or analyzed during the current study are available from the corresponding author on reasonable request.
